# Outcomes in Randomized Clinical Trials Testing Changes in Daily Water Intake: A Systematic Review

**DOI:** 10.1001/jamanetworkopen.2024.47621

**Published:** 2024-11-25

**Authors:** Nizar Hakam, Jose Luis Guzman Fuentes, Behnam Nabavizadeh, Architha Sudhakar, Kevin D. Li, Catherine Nicholas, Jason Lui, Peggy Tahir, Charles P. Jones, Stephen Bent, Benjamin N. Breyer

**Affiliations:** 1 Department of Urology, University of California, San Francisco; 2 Department of Urology, Weill Cornell Medicine, New York, New York; 3 UCSF Library, University of California, San Francisco; 4 Department of Medicine, University of California, San Francisco; 5 Department of Epidemiology and Biostatistics, University of California, San Francisco

## Abstract

**Importance:**

Several public recommendations exist regarding the amount of daily water intake, yet the supporting evidence is not clear, and benefits of increasing water consumption are not well-established.

**Objective:**

To summarize evidence from randomized clinical trials (RCTs) pertaining to the health-related outcomes associated with increased or decreased daily water consumption.

**Evidence Review:**

A systematic search of PubMed, Web of Science, and Embase was performed up to April 6, 2023. Studies were included if they aimed to assess the impact of daily water consumption by any defined amount on any health-related outcome.

**Findings:**

Of 1464 records screened, 18 (1%) eligible studies were included in the review. Among eligible studies, 15 (83%) were parallel group RCTs, and 3 (16%) were crossover studies. Interventions in these studies consisted of a recommendation to alter the daily amount of water intake by a specific amount for a predefined period ranging between 4 days and 5 years, while the control groups were mostly asked to maintain their usual intake habits. The studies assessed various populations. Recurring primary end points included weight loss, fasting blood glucose level, headache, urinary tract infection, and nephrolithiasis. Consuming additional water was associated with greater weight loss (range, 44%-100% more than control conditions) and fewer nephrolithiasis events (15 fewer events per 100 participants over 5 years). Single studies suggested benefits related to migraine prevention, urinary tract infection, diabetes control, and hypotension. Ten studies (55%) reported at least 1 positive result, and 8 studies (44%) reported negative results.

**Conclusions and Relevance:**

This systematic review found that there is a limited number of clinical trials in the literature assessing the benefits of increasing water intake related to a large variety of health outcomes. While the quality and quantity of evidence is limited, a small number of studies suggested benefits of water intake on weight loss and nephrolithiasis, while single studies raised the possibility of benefits for patients with migraine, urinary tract infection, diabetes, and hypotension. Given the low cost and low adverse-effect profile of water, further well-designed studies should assess benefits in these specific conditions.

## Key Points

**Question:**

Have any benefits of changing daily water intake been established?

**Findings:**

In this systematic review of 18 randomized clinical trials, interventions of increased water intake (or decreased intake in 1 study) were associated with statistically significant greater weight loss and fewer nephrolithiasis events. Single studies suggested benefits related to migraine prevention, urinary tract infection, diabetes control, and hypotension but did not reach statistical significance.

**Meaning:**

While the quality and quantity of evidence in the literature was limited, a small number of studies suggested benefits of water intake on weight loss and nephrolithiasis, while single studies suggested benefits related to migraine prevention, urinary tract infection, diabetes control, and hypotension.

## Teaser

This systematic review summarizes randomized clinical trials that tested the impact of changing participants’ daily water intake on a number of health-related outcomes, including weight loss, headache, urinary tract infections, and nephrolithiasis.

## Introduction

Water is a major constituent of the human body and is considered an essential nutrient that cannot be sufficiently produced by metabolism.^1^ The National Academy of Medicine suggests a daily fluid intake of approximately 13 eight-ounce cups for men and 9 for women, respectively.^2^ A common public health-related recommendation is to drink 8 cups of water per day, yet the supporting evidence is not clear. Given the wide variability of body weight, activity level, and health status at the population level and the numerous mechanisms that regulate water balance, a single optimal amount of daily water consumption is a challenging concept.^3^

Behavioral factors and hydration status have been widely studied in relation to health conditions owing to the known detrimental effects of dehydration.^4,5,6,7,8,9,10^ To our knowledge, there has been no study to compile reports of the benefits of water interventions on clinical outcomes. The purpose of this systematic review is to summarize evidence from randomized clinical trials (RCTs) pertaining to the impact of increased daily water consumption on health-related outcomes.

## Methods

Our systematic review followed an a priori registered protocol (CRD42021258697) and conformed to the Preferred Reporting Items for Systematic Reviews and Meta-Analyses (PRISMA) statement guidelines.^11^ We used data from published RCTs in which the original authors obtained informed consent from participants.

### Study Eligibility

Studies were eligible if their research question was to assess the impact of daily water consumption by any defined amount on any health-related outcome. Study design of interest was restricted to RCTs because our aim was to focus on reports of the causal effect of water consumption and to overcome the confounding bias that would be present in observational studies.

### Search Strategy

We searched PubMed, Web of Science, and Embase from inception to April 6, 2023. We developed search strategies in consultation with a research librarian (P.T.). In brief, our searches were developed around 2 main topics: water consumption and health-related issues. For each topic, multiple synonyms were included such as daily *water intake* or *water consumption* (topic 1) and *urinary symptoms* or *headache* (topic 2). Additional synonyms were developed besides these examples. Full search strategies for each database are in eAppendix 1 in Supplement 1. Manual screening of citations from articles selected for data extraction was also performed.

### Study Selection

Titles and abstracts of each of the search results were screened by 2 reviewers (N.H., J.L.G.F., B.N., A.S., K.D.L., C.N., and J.L.) independently and in duplicate. Full texts for citations identified as eligible by at least 1 reviewer were obtained and screened by 2 reviewers who used a standardized form to record reasons for exclusion. Disagreements were resolved by referring to a senior investigator (C.P.J. and B.N.B.). Results of the selection process were summarized using a PRISMA flow diagram (Figure). Two of the reviewers independently abstracted the following data: type of clinical trial, year of publication, description of study target population, description of intervention and comparator groups, primary end point assessed, results, and effect estimate (if reported).

**Figure.**
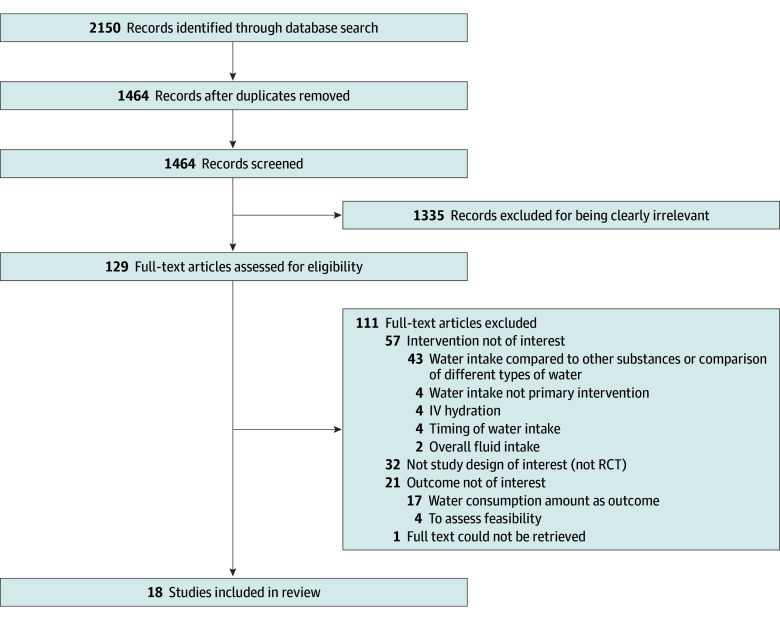
Flow Diagram for Study Selection Process IV indicates intravenous; RCT, randomized clinical trial.

### Data Analysis

Data were synthesized narratively by presenting descriptive data and summary results. A meta-analysis was not performed because the populations and outcomes assessed were not similar between studies. The Cochrane Risk-of-Bias tool for randomized studies (version 2) was used to assess the risk of bias in included studies.^12^

## Results

### Summary of Literature Search

Our search strategy yielded a total of 1464 unique studies that were screened to finally include 18 RCTs^13,14,15,16,17,18,19,20,21,22,23,24,25,26,27,28,29,30^ published between 1999 and 2023 (Figure). Of these, 15 (83%)^13,16,17,18,19,20,21,22,23,24,25,26,27,28,30^ were parallel group RCTs, and 3 (16%)^14,15,29^ were crossover studies. The median sample size was 48 participants and ranged between 14 and 631 participants. Interventions in these studies consisted of a recommendation to alter the daily amount of water intake by a specific amount for a predefined period, while the control groups were mostly asked to maintain their usual intake habits. Duration of the increased water intake intervention ranged between 4 days and 5 years. The studies assessed various populations and primary end points. In total, 10 studies (55%)^13,14,15,16,17,18,19,20,21,22^ reported at least 1 positive result and 8 studies (44%)^23,24,25,26,27,28,29,30^ reported negative results. Characteristics and summaries of positive and negative results in studies are depicted in the Table. Using the Cochrane risk of bias tool, 9 studies (50%)^16,18,21,23,24^ had a low risk of bias, 5 (27%)^13,14,19,20,26^ had some concern for bias, and 4 (22%)^15,17,22,25^ had high risk for bias (eAppendix 2 in Supplement 1). Common sources of bias were related to the allocation sequence randomization and concealment and loss to follow-up.

**Table.** Summary of Findings From Included Randomized Clinical Trials

Source	Population	No. randomized	Intervention^a^	Duration	Primary end point	Summarized results
**Weight loss (4 studies)**							
Wong et al,^28^ 2017	Adolescents with overweight or obesity who drink ≤4 cups/d	38	Increase water intake to 8 eight-oz cups/d (1920 mL)	6 mo	6-mo Change in BMI *z* score	Effect size, −0.0 (95% CI, −0.1 to 0.1; *P* = .88)
Parretti et al,^18^ 2015^b^	Adults with BMI ≥30	84	500 mL of water 30 min before main meals^c^	12 wk	Weight change	Greater weight loss (1.3 [95% CI, 0.14 to 2.4] kg; *P* = .03) in intervention vs control group
Akers et al,^20^ 2012^b^	Adults with overweight or obesity	39	Hypocaloric diet with premeal water (1420 mL)^d^	12 mo	Weight change	87% Greater weight loss (β = −0.01; *P* < .01) in intervention vs control group
Dennis et al,^19^ 2010^b^	Adults with overweight or obesity	48	Hypocaloric diet with 500 mL water prior to each of 3 daily meals^d^	12 wk	Weight change	44% Greater weight loss (β = −0.60; *P* < .001) in intervention vs control group
**FBG (2 studies)**							
Sedaghat et al,^16^ 2021^b^	Adults with type 2 diabetes	40	Increase premeal water by 1000 mL/d	8 wk	FBG after 8 wk	Significant between-group difference in change in FBG in intervention (mean, −32.6 mg/dL) vs control (mean, 5.3 mg/dL) (*P* = .003)
Nakamura et al,^26^ 2020	Adults with FBG level 90-126 mg/dL	60	1100 mL/d additional water intake	12 wk	FBG	Change in FBG approximately 0.6 mg/dL in both groups (*P* = .87)
**Headache (2 studies)**							
Spigt et al,^17^ 2012^b^	Adults with primary recurrent headache	102	1500 mL/d additional water intake	3 mo	MSQL score and days with at least moderate headache per month	Intervention associated with increased MSQL (4.5 [95% CI, 1.3 to 7.8] points; *P* = .007); intervention group had fewer days with at least moderate headache (−0.17 [95% CI −1.3 to 1.6] days; *P* = .82)
Spigt et al,^25^ 2005	Adults with migraine or tension headache for past 6 mo	18	1500 mL/d additional water intake	12 wk	MSQL score and headache measures	MSQL, −1 (95% CI, −9 to 7) points; medication use, −1 (95% CI, −6 to 4) tablets; episodes −1 (95% CI, −4 to 2); mean headache intensity, −13 (95% CI, −32 to 5) points; and headache time, −21 (95% CI, −48 to 5) hr
**Urinary tract infection and overactive bladder (3 studies)**							
Vento et al,^29^ 2023	Premenopausal women <1.5 L/d fluid intake	14	1900 mL/d water intake	4 d After menstruation begins	Urine culture positivity for pathogens	2 of 26 Positive cultures (7%) in intervention vs 2 of 23 (8%) in the control group
Hooton et al,^21^ 2018^b^	Premenopausal women with recurrent cystitis	140	1500 mL/d additional water intake	12 mo	Frequency of recurrent cystitis over 12 mo	Associated with fewer episodes (1.5 [95% CI, 1.2 to 1.8] episodes; *P* < .001)
Hashim et al,^15^ 2008^b^	Adults with overactive bladder	24	4 d Drinking 25% <baseline, and 2 d normal, then 4 d at 50% <baseline, and 2 d normal, then 4 d at 25% >baseline, and 2 d normal, and then 4 d at 50% >baseline^e^	3 wk	Frequency of unwanted events (day and night voids, urgency and urgency incontinence episodes) during a 24 h period	Significantly reduced daytime frequency, urgency, and nocturia in the intervention groups that decreased intake
**Nephrolithiasis (2 studies)**							
de La Guéronniere et al,^13^ 2011^b^	Healthy adults	48	2000 mL/d additional water intake	6 d	Stone risk assessment measured by Tiselius CRI	Tiselius CRI decreased in intervention group (mean [SD] 1.01 [0.74] to 0.63 [0.45]) but increased in the control group (0.84 [0.37] to 1.29 [1.06])
Borghi et al,^22^ 1996^b^	Patients with first episode of idiopathic calcium nephrolithiasis	221	High water intake to achieve urine volume of ≥2000 mL/d	5 y	Frequency of stone events and mean time to event	Recurrence rate was lower in intervention group (12 of 99) than control group (27 of 100) (*P* = .008); mean (SD) time to recurrences was longer in intervention (38.7 [13.2] mo) vs control (25.1 [16.4] mo) (*P* = .02)
**Other outcomes (5 studies)**							
Clark et al,^30^ 2018	Adults with stage 3 chronic kidney disease	631	Increase oral water intake by 1000-1500 mL/d	12 mo	eGFR at 12 mo	Effect size, −0.3 (95% CI, −1.8 to 1.2; *P* = .74)
Jimenez et al,^24^ 2015	Male smokers (moderate to heavy) age 20-45 y	65	1500 mL/d additional water intake	50 d	4-ABP and BPDE-DNA adducts formation in exfoliated bladder cells in 24 h urine	Adjusted mean change (SE), −0.068 (0.127) vs −0.042 (0.127); *P* = .89 for 4-ABP-DNA; −0.131 (0.144) vs −0.054 (0.144), *P* = .71 for BPDE-DNA
Sontrop et al,^23^ 2015	Adults with stage 3 chronic kidney disease	29	Increase oral water intake by 1000-1500 mL/d	6 wk	Plasma copeptin at 6 wk	Median change −3.6 pmol/L in intervention vs −1.1 pmol/L in control group (*P* = .19)
Jormeus et al,^14^ 2010^b^	Healthy individuals	20	Additional 30 mL water/kg body weight/d	2 wk	Ambulatory BP	Increased mean (SD) arterial daytime BP from 89.0 (5.5) to 91.4 (6.4) mm Hg (*P* = .005), night-time BP unchanged
Spigt et al,^27^ 2006	Older (age 55-75) men	141	Advised to drink additional 1500 mL/d of water	6 mo	Blood sodium, GFR, BP, SF-36 quality of life score	Outcomes associated with intervention: sodium level, −0.2 (95% CI, −1.1 to 0.6) mmol/L; GFR, −0.1 (95% CI, −3.2 to 2.9) mL/min/1.73 m^2^; systolic BP −2.4 (95% CI, −8.0 to 3.2) mm Hg; diastolic BP −0.9 (95% CI, −3.7 to 2.0) mm Hg; SF-36 1.1 (95% CI, −2.4 to 4.8) points

Abbreviations: ABP, aminobiphenyl; BMI, body mass index (calculated as weight in kilograms divided by height in meters squared); BP, blood pressure; BPDE, benzo[a]pyrene diol epoxide; CRI, crystallization risk index; eGFR, estimated glomerular filtration rate; FBG, fasting blood glucose; MSQL, Migraine Specific Quality of Life; SF-36, 36-item Short Form.

SI conversion factors: To convert FBG to millimoles per liter, multiply by 0.0555; sodium to milliequivalents per liter, divide by 1.

^a^ Comparison groups were instructed to maintain usual water intake unless otherwise indicated.

^b^ Indicates a randomized clinical trial with a positive result.

^c^ Participants in comparison group were asked to imagine their stomach was full before meals.

^d^ Participants in comparison group were given a hypocaloric diet only.

^e^ Comparison was reverse order of intervention groups, ie, drinking more, then drinking less.

### End Points Assessed

#### Weight Loss

Four studies (22%)^18,19,20,28^ evaluated the effect of water on weight change in participants with overweight and obesity. In 3 parallel group RCTs,^18,19,20^ adults with overweight and obesity randomized to consume 1500 mL of water per day before meals for a period of 12 weeks to 12 months had greater weight loss compared with those in the control groups (approximately 100%, 87%, and 44% more weight vs control groups).

In a fourth study^28^ including 38 participants who reported drinking less than 1000 mL/d, drinking 2000 mL/d was not associated with weight over a 6-month period (effect estimate, 0.0 [95% CI, −0.1 to 0.1]; *P* = .88). The study recruited only 63% of its target, and at 6 months, the intervention group reported drinking 1135 (95% CI, 900 to 1400) mL/d. Compared with the other 3 studies assessing weight change, this study was distinct because it included adolescents aged 17 years or younger, whereas the other studies included adults only, and the water intervention in this study was not premeal intake.

#### Fasting Blood Glucose

Two studies (11%)^16,26^ assessed the effect of a water intervention on fasting blood glucose (FBG) levels. Sedaghat et al^16^ randomized 40 patients with recently diagnosed (<5 years) type 2 diabetes into a water intervention or control group that received no water recommendation. The water intervention consisted of drinking 250 mL before breakfast, 500 mL before lunch, and 250 mL before dinner for a total of 8 weeks. There was a significant between-group difference in mean change in FBG in the intervention (−32.6 mg/dL) vs the control group (5.3 mg/dL) (*P* = .003) (to convert to millimoles per liter, multiply by 0.0555). On the other hand, Nakamura et al^26^ tested a water intervention consisting of drinking 550 mL within 2 hours of waking and 550 mL before bedtime and found that FBG increased by approximately 0.6 mg/dL after 12 weeks in both groups.^26^ This RCT consisted of 60 adults with baseline FBG between 90 and 126 mg/dL and excluded participants receiving medication and/or lifestyle modification for diabetes. Baseline FBG of study participants was approximately 97 mg/dL, substantially lower than that in Sedaghat et al,^16^ which was approximately 225.7 mg/dL in the intervention group and 221.7 mg/dL in the control group.

#### Headache

Two studies (11%)^17,25^ assessed increasing water intake by 1500 mL/d for 3 months in patients with recurrent headaches with conflicting results. One study^17^ recruited 102 adults with primary recurrent headaches and found that the intervention was associated with improvements on their Migraine Specific Quality of Life (MSQL) score (range 0-100) by 4.5 (95% CI, 1.3 to 7.8) points (*P* = .007).^17^ Participants in the intervention group experienced fewer days with at least moderate headache (0.17 [95% CI, −1.3 to 1.6] days; *P* = .82), and more patients in the intervention group reported much improvement (effect estimate, 2.6 [95% CI, 1.0 to 6.8]; *P* = .05) compared with the control group, but these findings were not statistically significant. Many patients did not complete the study (21% and 34% in the intervention and control group, respectively). Another study^25^ used the same intervention and included 18 adults with migraine or tension headache, and the effect of the intervention was not statistically significant on MSQL (−1 [95% CI, −9 to 7] points), medication use (−1 [95% CI, −6 to 4] tablets), number of episodes (−1 [95% CI, −4 to 2]), mean headache intensity (−13 [95% CI, −32 to 5] points), or hours of headache (−21 [95% CI, CI −48 to 5] hours).^25^ However, the sample size was low and the confidence intervals allow for a wide range of improvement or decline.

#### Urinary Tract Infection and Overactive Bladder

In an RCT of 140 premenopausal women with recurrent urinary tract infections (UTI) who drank less than 1500 mL fluid/d,^21^ increasing water intake by 1500 mL/d was associated with a lower mean number of UTI episodes (1.5 [95% CI, 1.2-1.8]; *P* < .001) over a 12-month period. The intervention was also associated with a lower mean number of antimicrobial regimens used to treat UTI episodes (1.7 [95% CI, 1.3-2.1]; *P* < .001), improved median time to first UTI episode (hazard ratio, 0.51 [95% CI, 0.36-0.74]; *P* < .001), and increased mean interval between episodes (58.4 [95% CI, 39.4-77.4] days; *P* < .001).

Vento et al^29^ assessed increased water intake on uropathogenic bacterial activity in underhydrated premenopausal women. Fourteen women with a baseline average fluid intake of less than 1500 mL/d were randomized to a group instructed to drink 1900 mL water per day for 4 days starting on the second day of a menstrual cycle. The control group was instructed to maintain usual habits. During the following menstrual cycle, participants in the intervention group switched to the control group and vice versa. First-morning urine samples were collected for culture on days 2, 3, 5, and 6 of menstruation. The rate of pathogen positive cultures was similar between groups (7% in the intervention vs 8% in the control group).

Another study^15^ randomized 24 adults with symptoms of overactive bladder to either increasing or decreasing their fluid intake in a crossover RCT. Decreasing fluid intake by 25% was associated with reduced frequency, urgency, and nocturia.

#### Nephrolithiasis

Two studies (22%)^13,22^ examined increased water intake and kidney lithiasis risk. De La Guéronnière et al^13^ measured Tiselius crystallization risk indices (CRI) in 2 groups randomized to either 2000 mL additional water intake per day or a control group. Higher Tiselius CRI suggests higher risk of stone formation. Participants were healthy adults aged 25 to 50 years. Tiselius CRI declined in the intervention group but increased in the control group.^13^ Borghi et al^22^ randomized 221 patients after a first episode of idiopathic calcium nephrolithiasis into an increased water intake group (to achieve a urine volume 2000 mL/d) or a control group. Over 5 years, stone recurrence rate was significantly lower in the intervention group (12 of 99 participants) compared with the control group (27 of 100 participants) (*P* = .008), for approximately 15 fewer episodes per 100 participants over 5 years. Mean (SD) time to recurrence was significantly longer in the intervention group (38.7 [13.2] months) vs the control group (25.1 [16.4] months) (*P* = .02).

#### Other Outcomes

Patients with stage 3 chronic kidney disease^30^ were randomized into a 12-month intervention of additional water intake of 1000 to 1500 mL/d vs control group, and no statistically significant difference in glomerular filtration rates was found (effect estimate, −0.3 [95% CI, −1.8 to 1.2] mL/min/1.73 m^2^; *P* = .74). One study^24^ assessed DNA adducts formation in exfoliated bladder cells, regarded as a precursor of bladder cancer, and found no difference between the intervention and control group. Another study^23^ found no effect of a 6-week water intervention on plasma copeptin (a marker of vasopressin, where higher levels predict kidney function decline) level in patients with stage 3 chronic kidney disease. In 20 healthy individuals without hypotension or hypertension,^14^ drinking an additional 30 mL water per kilogram of body weight per day was associated with increased daytime mean (SD) arterial blood pressure from 89.0 (5.5) to 91.4 (6.4) mm Hg (*P* = .005) while night-time blood pressure remained unchanged compared with the control group. Lastly, one study^27^ did not find a statistically significant association between increased daily water intake (by 1500 mL for 6 months) and blood sodium, blood pressure, glomerular filtration rate, or quality of life score (assessed by 36-Item Short Form Survey).

## Discussion

To our knowledge, this is the most comprehensive systematic review to assess the benefits of altered daily water intake interventions. Our study was restricted to RCTs to focus on the reported causal effects of water. We found that increased water intake (and decreased intake in one study) was statistically significantly associated with beneficial effects on several clinical outcomes. Some end points were assessed by more than 1 study and showed mixed results as to whether a statistically significant effect was detected.

### Weight Loss

Three studies documented greater weight loss in adult participants with an intervention of premeal water intake of approximately 500 mL.^18,19,20^ Premeal water intake may induce weight loss through stomach filling, leading to earlier satiety or by replacing high-calorie beverages. In a study by Van Walleghen et al,^31^ premeal water consumption (500 mL) was shown to reduce meal energy intake in older adults by approximately 111 kcal. Interestingly, this outcome was not detected in younger adults in this study. Additionally, subjective hunger ratings were lower, while subjective fullness ratings were higher with premeal water consumption.^31^ Davy et al^32^ conducted a study in which 24 adults with overweight or obesity with a mean (SD) age of 61.3 (1.1) years were given a standard breakfast meal on 2 randomly assigned occasions, only 1 preceded by a 500-mL water preload.^32^ They found a statistically significant 13% reduction in meal energy intake with water preload. A psychosocial benefit may also exist, in which water intake might serve as a reminder to eat healthier food.

Establishing a simple, low-cost intervention (eg, premeal water) for weight loss can be enormously beneficial for public health given the increasing health burden of obesity.^33^ For example, in the United States, obesity prevalence increased from 30.5% to 41.9% over the past 2 decades, and the estimated medical cost of obesity was nearly $173 billion dollars in 2019.^34^ Additionally, a significant portion of vascular deaths (29%) and neoplastic deaths (8%) in late middle age in the United States were attributable to elevated body mass index.^33^

Another study^28^ involving 38 adolescent participants failed to demonstrate a beneficial association of a water intervention with weight loss over a 6-month period using an intention-to-treat analysis. Despite not achieving their target for recruitment, the authors reported that the observed difference between groups would still not be statistically significant if recruitment was complete. Nevertheless, the target intake of 2000 mL/d was not achieved, with only 2 participants reporting drinking 1775 mL/d or more. As many as 40% of the participants in the intervention group reported not being able to drink in class or not having convenient or safe places to drink water, suggesting that adherence with the intervention is more difficult to achieve in an adolescent population.

### FBG

A premeal water intervention for a total of 1000 mL/d was significantly associated with improved FBG in patients with diabetes with baseline FBG between 220 and 230 mg/dL over an 8-week period.^16^ The magnitude of decrease in FBG was relatively large and seems clinically important. Given the high prevalence of diabetes, future studies could further investigate this as a low-cost treatment option to avoid medication use or escalation. It is unclear whether this resulted from hemodilution. In Nakamura et al,^26^ patients with a normal baseline FGB level did not experience a decrease in FBG after a water intervention. It could be hypothesized that in those with already elevated FBG, increasing the plasma fluid volume can help normalize the glucose concentration when the total amount of glucose in the blood is already increased. One aspect of the water intervention differed between these 2 studies: in Sedaghat et al,^16^ patients were instructed to consume water before meals, whereas in Nakamura et al,^26^ water consumption was not specifically linked to mealtimes. Thus, the benefits observed in Sedaghat et al^16^ could be due to decreased food intake or weight loss. This is supported by the notion that in Sedaghat et al,^16^ participants in the intervention group experienced weight loss whereas those in the control group did not.

### Headache

Adults with primary recurrent headaches reported better quality of life after 3 months of increase water intake.^17^ The 2 available RCTs related to headache suggested that water may improve objective and subjective headache outcomes, as the effect estimates were mostly indicative of a beneficial effect for many outcomes with wide confidence intervals. We postulate that low sample size and high dropout rates contributed to the null outcomes. Prior investigations suggested a potential beneficial effect of increased water intake on migraine headache prevention and treatment; however, the quality of evidence is not robust and consists mostly of case reports.^35,36,37,38^ Future research is warranted to better understand whether there is a therapeutic or preventive role for water interventions for patients with recurrent or debilitating headaches.

### Urinary Tract Infection and Overactive Bladder

In women with recurrent UTIs and less than 1500 mL of fluid intake per day, increasing water intake by 1500 mL was associated with a reduced number of episodes and a longer duration between episodes.^21^ It is hypothesized that high fluid intake reduces UTI risk by several mechanism, including diluting and flushing bacteriuria, improving clearance, and inhibiting attachment to epithelial cells.^39,40,41,42,43^ Given the high prevalence of uncomplicated UTIs in young women, a simple intervention of increased water intake is a valuable and safe method to obviate, or reduce, antibiotic utilization and prevent antibiotic resistance. One study^29^ hypothesized a primary preventive role against UTI of a 1900 mL/d water intervention but could not demonstrate such an effect. In this study,^29^ urine cultures performed on days 2 to 5 of a menstrual cycle were compared between healthy women in the intervention and the control group, and culture positivity for urinary pathogens was similar between groups. However, there were several shortcomings to this analysis. First, the small sample of 14 participants randomized might not represent the population who would likely benefit from such an intervention if a benefit exists; 8 had no UTI history and 5 had only 1 prior infection. Additionally, the duration of the intervention was short; the intervention started on the second day of the menstrual cycle and lasted for 4 days, and urine samples for cultures were taken daily on those same days. Lastly, in the setting of a crossover RCT, being in the control group after already participating in the intervention group would make the participant more conscious about and might influence their water intake. This is opposite to a study in which those in the control group were blinded to the actual goal of the study.

Decreasing fluid intake by 25% in adults with overactive bladder symptoms significantly reduced frequency, urgency, and nocturia. Fluid manipulation is a noninvasive and safe method and should be considered in most cases of patients with overactive bladder.

### Nephrolithiasis

Two RCTs in the literature^13,22^ addressed nephrolithiasis-related end points and increased water intake. Increasing water intake to achieve a daily urine output of 2000 mL or more after an idiopathic calcium stone was associated with significantly fewer stone events by more than half; this intervention was also associated with increased time to recurrence.^22^ It is also suggested that increasing water intake in healthy individuals decreases stone risk, judged by an improved Tiselius CRI.^13^ Urinary concentration of stone forming salts is an essential part of kidney stone formation. Altering urine volume can thus interfere with these concentrations and prevent stone formation by preventing supersaturation. Stone formers are generally recommended to increase their fluid intake to achieve a urine volume of at least 2.5 L/d.^44^

### Other Outcomes

RCTs of interventions involving increased daily water intake found that increased intake was associated with increased mean daytime arterial blood pressure in young adults with hypotension. Water intake interventions did not seem to affect glomerular filtration rate or plasma copeptin in patients with stage 3 chronic kidney disease, DNA adducts formation in exfoliated bladder cells, or any of blood sodium, blood pressure, glomerular filtration rate, or quality of life scores in older men.

Several factors contribute to water requirements, and water content from food intake is often overlooked and difficult to calculate. In a study of more than 5600 individuals aged between 8 and 96 years from 23 countries,^45^ isotope tracking methods were used to assess water turnover and found that numerous factors (eg, age, body size, activity and athleticism, environmental and geographical factors, socioeconomic status, and others) are all associated with water turnover, which is widely variable. In this systematic review, we found that not only interventions that increased water intake but also those that decreased water intake were associated with beneficial outcomes according to patient status and preexisting conditions. We also found that these interventions might produce no effect for other end points. These findings further the argument that water intake should ideally be individualized and that a recommendation for a single optimal daily consumption amount is challenging.

### Limitations

There are some limitations to our findings. Mainly, the results of RCTs are interpreted from an intention-to-treat perspective; thus, the actual causal effect of water might not have been accurately demonstrated. While interventions consisted of a recommendation for increasing water intake, adherence to these recommendations and actual intake are not guaranteed. Moreover, since observational studies on the topic may be heavily confounded and subject to several biases, especially recall bias, we restricted our study to RCTs that led to fewer included studies. Lastly, many end points were examined by single studies and thus only limited evidence is available for these end points. The literature would benefit from further studies confirming these results, especially in cases in which the primary end point is a clinical outcome and when the current available studies have obvious limitations or could not achieve target recruitment or participants did not adhere to the intervention. Although current RCTs specify a certain volume of water as the intended intervention, they do not establish what the optimal or target total daily intake should be, which could be addressed in future studies.

## Conclusions

This systematic review found that there is a limited number of clinical trials in the literature assessing the benefits of increasing water intake related to a large variety of health outcomes. While the quality and quantity of evidence was limited, a small number of studies suggest benefits of water intake on weight loss and nephrolithiasis, while single studies raise the possibility of benefits related to migraine prevention, UTIs, diabetes control, and hypotension. Given the obvious low cost and low adverse-effect profile of water, further well-designed studies should assess benefits in these specific conditions.

## Notes

Supplement 1. **eAppendix 1.** Search Strategy **eAppendix 2.** Risk of Bias Assessment Results Using the Cochrane Risk-of-Bias Tool for Randomized Trials (Version 2)

Supplement 2.
Data Sharing Statement
