# Supplementary material for: Outcomes in Randomized Clinical Trials Testing Changes in Daily Water Intake: A Systematic Review
Source: JAMA Netw Open. 2024 Nov 25;7(11):e2447621. doi: 10.1001/jamanetworkopen.2024.47621 (PMC11589796; doi:10.1001/jamanetworkopen.2024.47621)
Supplement: Supplement 1. — eAppendix 1. Search Strategy eAppendix 2. Risk of Bias Assessment Results Using the Cochrane Risk-of-Bias Tool for Randomized Trials (Version 2) [file jamanetwopen-e2447621-s001.pdf]

Supplemental Online Content

Hakam N, Guzman Fuentes JL, Nabavizadeh B, et al. Outcomes in randomized clinical trials testing changes in daily water intake. *JAMA Netw Open.* 2024;7(11):e2447621. doi:10.1001/jamanetworkopen.2024.47621

eAppendix 1. Search Strategy

eAppendix 2. Risk of Bias Assessment Results Using the Cochrane Risk-of-Bias Tool for Randomized Trials (Version 2)

This supplemental material has been provided by the authors to give readers additional information about their work.

© 2024 Hakam N et al. *JAMA Network Open.*

eAppendix 1. Search Strategy

Database	Search Strategy
PubMed	("daily water intake" OR "water intake" OR "water supplementation" OR "extra water" OR "water consumption" OR "water drinking" OR "drinking water" OR “Drinking Water”[Mesh] OR "drinking more water" OR "water needs" OR “increased water” OR ((“fluid intake” OR “fluid input” OR “increased fluid” OR "Drinking Behavior”[Mesh]) AND water)) AND (hydration OR "Organism Hydration Status"[Mesh] OR health[tiab] OR “Health”[mesh] OR healthy[tiab] OR “Health Behavior”[Mesh] OR headache OR headaches OR “Headache”[Mesh] OR "Urinary Incontinence"[Mesh] OR “urinary symptoms” OR "Urinary Tract Infections"[Mesh] OR “urinary tract infections” OR cystitis OR "Diet, Reducing”[Mesh] OR overweight OR “Overweight”[Mesh] OR obesity OR “Obesity”[Mesh] OR “weight loss”) AND (Therapy/Narrow[filter])
Web of Science	("daily water intake" OR "water intake" OR "water supplementation" OR "extra water" OR "water consumption" OR "water drinking" OR "drinking water" OR "drinking more water" OR "water needs" OR “increased water” OR ((“fluid intake” OR “fluid input” OR “increased fluid” OR "drinking behavior”) AND water)) AND (hydration OR health OR healthy OR “health behavior” OR headache OR headaches OR "urinary incontinence" OR “urinary symptoms” OR “urinary tract infections” OR cystitis OR "reducing diet” OR overweight OR obesity OR “weight loss”) AND (“randomized controlled trial” OR “randomized controlled trials” OR RCT OR RCTs OR “randomised controlled trial” OR “randomised controlled trials”)
Embase	('daily water intake' OR 'water intake'/exp OR 'water intake' OR 'water supplementation' OR 'extra water' OR 'water consumption'/exp OR 'water consumption' OR 'water drinking'/exp OR 'water drinking' OR 'drinking water'/exp OR 'drinking water' OR 'drinking more water' OR 'water needs' OR 'increased water' OR (('fluid intake'/exp OR 'fluid intake' OR 'fluid input' OR 'increased fluid' OR 'drinking behavior'/exp OR 'drinking behavior') AND ('water'/exp OR water))) AND (hydration OR 'hydration status' OR health:ab,ti OR healthy:ab,ti OR 'health behavior' OR headache OR 'urine incontinence' OR 'urinary symptoms' OR 'urinary tract infection' OR cystitis OR 'low calorie diet' OR obesity OR overweight OR 'body weight loss') AND ('randomized controlled trial'/exp OR 'randomized controlled trial' OR 'randomized controlled trial (topic)'/exp OR 'randomized controlled trial (topic)' OR rct OR rcts)

© 2024 Hakam N et al. *JAMA Network Open.*

eAppendix 2. Risk of Bias Assessment Results Using the Cochrane Risk-of-Bias Tool for Randomized Trials (Version 2)

Study	D1	D2	D3	D4	D5	Overall
Wong 2017	+	+	+	+	+	+
Parretti 2015	+	+	+	+	+	+
Akers 2012	!	+	+	+	+	!
Dennis 2010	!	+	+	+	+	!
Sedaghat 2021	+	+	+	+	+	+
Nakamura 2020	!	+	+	+	+	!
Spigt 2012	+	+	-	+	+	-
Spigt 2005	+	+	-	+	+	-
Vento 2023	+	+	+	+	+	+
Hooton 2018	+	+	+	+	+	+
Hashim 2008	!	+	+	-	+	-
de La Guéronniere 2011	!	+	+	+	+	!
Borghi 1999	!	+	-	+	+	-
Clark 2018	+	+	+	+	+	+
Jimenez 2015	+	+	+	+	+	+
Sontrop 2015	+	+	+	+	+	+
Jormeus 2010	!	+	+	+	+	!
Spigt 2006	+	+	+	+	+	+

+ Low risk

! Some concerns

- High risk

D1 Randomisation process

D2 Deviations from the intended interventions

D3 Missing outcome data

D4 Measurement of the outcome

D5 Selection of the reported result

© 2024 Hakam N et al. *JAMA Network Open.*
