# Supplementary material for: Outcomes in Randomized Clinical Trials Testing Changes in Daily Water Intake: A Systematic Review
Source: JAMA Netw Open. 2024 Nov 25;7(11):e2447621. doi: 10.1001/jamanetworkopen.2024.47621 (PMC11589796; doi:10.1001/jamanetworkopen.2024.47621)
Supplement: Supplement 2. — Data Sharing Statement [file jamanetwopen-e2447621-s002.pdf]

Data Sharing Statement

Hakam. Outcomes in Randomized Clinical Trials Testing Changes in Daily Water Intake. *JAMA Netw Open*. Published November 25, 2024. doi:10.1001/jamanetworkopen.2024.47621

Data
Data available: No

Additional Information
Explanation for why data not available: All data used in this manuscript is published in the literature and thus already available. We made every effort to cite all sources of information.
